# Association between Parkinson's disease and risk of colorectal cancer: A systematic review and meta‐analysis

**DOI:** 10.1002/ibra.12193

**Published:** 2025-01-22

**Authors:** Meng‐Dan Su, Tian‐Hong Wang, Hao‐Wen Zhang, Ke‐Yu Cao, Fei Liu

**Affiliations:** ^1^ Department of Anesthesiology West China Hospital, Sichuan University Chengdu China; ^2^ Department of Thoracic Surgery West China Hospital, Sichuan University Chengdu China; ^3^ Department of Nursing West China Hospital, Sichuan University/West China School of Nursing, Sichuan University Chengdu China

**Keywords:** colorectal cancer, meta‐analysis, Parkinson's disease, pooled analysis, systematic review

## Abstract

This study aims to investigate the relationship between Parkinson's disease (PD) and colorectal cancer (CRC) risk by a systematic review and meta‐analysis. Using Embase, Pubmed, and Cochrane Library databases, 21 articles reporting clinical data of 1,635,873 PD patients and 10,388,842 healthy individuals were finally included. Based on the results of pooled analysis, we found that PD patients exhibited a decreased risk of CRC (relative risk (RR) = 0.74; 95% confidence interval (CI), 0.68–0.80). In contrast to case‐control (RR = 0.80; 95% CI, 0.64–1) and cohort studies (RR = 0.72; 95% CI, 0.66–0.79), the combined risk of PD patients with CRC in Asian nations (RR = 0.67; 95% CI, 0.58–0.78) was lower than that in Western countries (RR = 0.76; 95% CI, 0.70–0.82). In comparison to rectal cancer (RR = 0.82; 95% CI, 0.69–0.97), PD patients exhibited a lower combined risk of colon cancer (RR = 0.76; 95% CI, 0.67–0.86). Furthermore, the combined CRC risks for patients in studies published before 2010 and after 2010 were 0.76 (RR = 0.76; 95% CI, 0.66–0.88) and 0.74 (RR = 0.74; 95% CI, 0.68–0.80), respectively. These findings indicate that patients with PD had a reduced risk of CRC. Future studies are merited in exploring pathological molecular linkages or underlying mechanisms of inverse association between CRC and PD.

## Graphical abstract

The aim of this study is to examine the association between Parkinson's disease (PD) and colorectal cancer (CRC) risk through a systematic review and meta‐analysis. This study represents the largest meta‐analysis conducted to date on the relationship between CRC risk and PD. Our findings suggest that individuals with PD may have a reduced risk of developing CRC. Further research is warranted to investigate the potential pathological molecular pathways or underlying mechanisms that may explain the inverse association between CRC and PD.

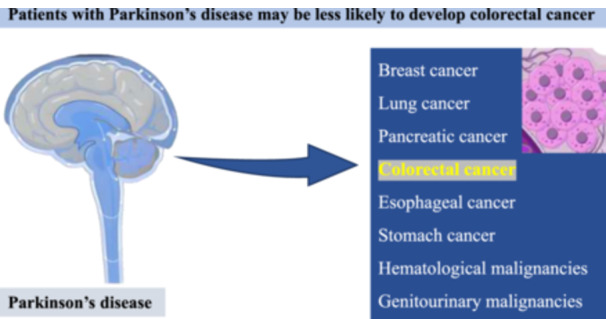

## 1 INTRODUCTION

Parkinson's disease (PD) is a recognizable clinical syndrome with a variety of causes and clinical manifestations.^1^ PD has placed a substantial burden on society as it affected 6.1 million people worldwide in 2016, up from 2.5 million in 1990.^2^ According to the study, 1.37% of over 60 years old had PD, suggesting that approximately 3.62 million people in China may have PD.^3^ PD may be difficult to detect in its earliest stages, as reflected by the long delay (average 10 years) between the first symptom and the diagnosis. Tremor at rest, muscle rigidity, akinesia (or bradykinesia), and postural instability, abbreviated as TRAP, are the four cardinal symptoms of PD.^4^ Classic symptoms of parkinsonism include freezing (motor blocks) and a flexed posture, with PD being the most common kind. Non‐motor symptoms include issues with perception, sleep patterns, and neurobehavioral and cognitive abnormalities.^4^ Therefore, PD significantly impacts the quality of life for older patients.

Interestingly, epidemiological studies revealed that while PD patients have increased chances of suffering from brain cancer and melanoma, they are less likely to suffer from many common types of cancer, such as hematological, gastrointestinal, lung, and genitourinary malignancies.^5^, ^6^, ^7^ The underlying mechanisms remain poorly understood. A study of 62,023 PD patients in China found that PD was linked to an increased risk of malignant brain tumors, gastrointestinal tract cancers, and lung cancers.^8^ Another study conducted in Korea demonstrated inverse associations between overall cancer incidence and various types of cancer in individuals with PD. The implications of these findings and their underlying mechanisms warrant additional research.^9^

Colorectal cancer (CRC) is a type of cancer that affects the colon (large intestine) or rectum, and it is the third most frequent cancer around the world. In 2020, it was estimated that there were 1.9 million new cases of CRC and 930,000 fatalities. Europe, New Zealand, and Australia had the highest incidence rates of CRC.^10^ Interestingly, a study conducted in Korea, which included 260,045 individuals without PD and 52,009 patients diagnosed with PD between 2010 and 2015, found that patients with PD had a decreased risk of CRC.^11^ Additionally, a recent study that examined populations from 183 countries indicated that nations with high PD mortality had a reduced probability of CRC mortality.^12^ As of now, however, the phenomena lack a well‐established explanation, and there is still conflicting evidence linking PD to CRC as an additional investigation revealed no connection between CRC and PD.^13^ Furthermore, epidemiological research on the link between CRC and PD confronts a variety of methodological problems and probable biases, encompassing diagnostic bias, confounding, conflicting hazards, or selective survival. Moreover, recent studies on the connection between CRC and PD have yielded mixed results. Therefore, this work aims to further explore any potential links between PD and the risk of CRC, addressing the existing uncertainties and providing new insights into this area of research.

## 2 METHODS

Meta‐analysis was implemented by applying Preferred Reporting Items for Systematic Reviews and Meta‐Analyses guidelines.^14^

### 2.1 Search strategy and selection

By searching EMBASE, PubMed, and Cochrane Library with the terms “Parkinson's disease,” “colon cancer,” “rectal cancer,” and “colorectal cancer,” relevant studies published in English up to January 2024 were retrieved. Additionally, we manually reviewed the bibliographies of all relevant articles to ensure no significant studies were missed. The databases were independently searched by two researchers, each oblivious to the other's findings to minimize bias. In the event of a disagreement, the results were reviewed and resolved by a third researcher to reach a consensus. The detailed search strategy for Pubmed database was as follows: (“parkinson disease”[MeSH Terms] OR (“parkinson”[All Fields] AND “disease”[All Fields]) OR “parkinson disease”[All Fields] OR “parkinson s disease”[All Fields]) AND (“colonic neoplasms”[MeSH Terms] OR (“colonic”[All Fields] AND “neoplasms”[All Fields]) OR “colonic neoplasms”[All Fields] OR (“colon”[All Fields] AND “cancer”[All Fields]) OR “colon cancer”[All Fields] OR (“rectal neoplasms”[MeSH Terms] OR (“rectal”[All Fields] AND “neoplasms”[All Fields]) OR “rectal neoplasms”[All Fields] OR (“rectal”[All Fields] AND “cancer”[All Fields]) OR “rectal cancer”[All Fields]) OR (“colorectal neoplasms”[MeSH Terms] OR (“colorectal”[All Fields] AND “neoplasms”[All Fields]) OR “colorectal neoplasms”[All Fields] OR (“colorectal”[All Fields] AND “cancer”[All Fields]) OR “colorectal cancer”[All Fields])).

### 2.2 Eligibility criteria

The inclusion criteria for this study centered on the relationship between the risk of CRC and PD. Studies that reported relative risk (RR), odds ratio (OR), or hazard ratio (HR) data were included, encompassing various study designs such as cohort studies, case‐control studies, and randomized controlled trials. Exclusion criteria encompassed studies with insufficient data to investigate these ratios, as well as meta‐analyses, reviews, letters, editorials, and case reports. Additionally, duplicate studies were excluded from the analysis.

### 2.3 Data extraction and quality assessment

Two authors independently collected the following data from the qualified research studies: author, year, nation, research design, number of control groups and cases, sex, age, follow‐up, tumor location, as well as outcomes. The quality of the included studies was evaluated according to the Newcastle‐Ottawa scale (NOS).^15^ The scale ranges from zero to nine stars: studies with scores higher than or equal to five are considered methodologically sound. It was performed independently by two researchers to reduce bias and risk. In cases where discrepancies arose, a third reviewer was consulted to resolve the differences.

### 2.4 Statistical analysis

The association between CRC risk and PD was assessed utilizing pooled RR and matching 95% confidence intervals (CIs) based on selnRR and lnRR. Heterogeneity between the studies was evaluated through the *I* ^2^ statistical approach and *Q*‐test to determine variation not solely caused by chance. *I* ^2^ ≥ 75%, 50% < *I* ^2^ ≤ 75%, 25% < *I* ^2^ ≤ 50%, and *I* ^2^ ≤ 25% indicated considerable, substantial, moderate, and slight heterogeneity, respectively. Subgroup analyses were performed based on countries, study design, year of publication, and tumor location. Sensitivity analysis was conducted on each research individually to ensure the stability of the results. A publication bias assessment was carried out utilizing Begg's test and funnel plots. A statistically significant result was defined as *p* < 0.05, and STATA version 16.0 was exploited for all statistical analyses.

## 3 RESULTS

### 3.1 Basic characteristics

**FIGURE 1:**
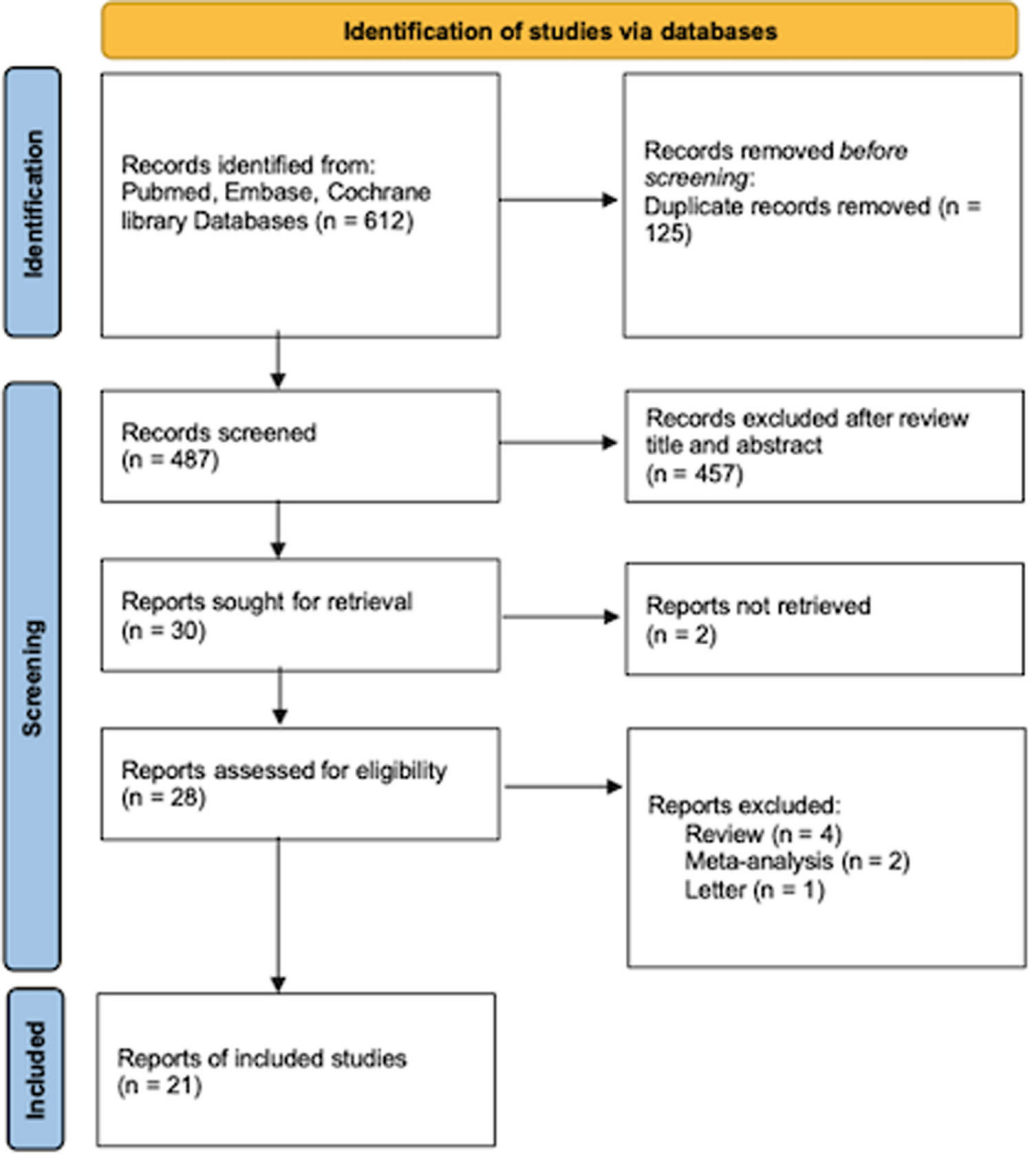
Flow chart of selection studies and specific reasons for exclusion. [Color figure can be viewed at wileyonlinelibrary.com]

Based on the search strategies, a total of 612 literatures were retrieved. After an initial review of the abstracts and titles, 28 papers were selected for further assessment. Of these, 21 articles^5^, ^8^, ^9^, ^11^, ^12^, ^13^, ^16^, ^17^, ^18^, ^19^, ^20^, ^21^, ^22^, ^23^, ^24^, ^25^, ^26^, ^27^, ^28^, ^29^, ^30^ were included in the final analysis, which provided relevant data on the relationship between PD and CRC. These studies involved a total of 1,635,873 PD patients and 10,388,842 healthy individuals. The flow chart of the study selection process is presented in Figure 1. Of the included studies, 16 studies were conducted in Western countries and 5 studies were from Asian countries. Furthermore, 17 studies were cohort studies, and four studies were case‐control studies. Seven studies were published in 2010 or earlier, and 14 studies were published after 2010. There has been evidence from nine studies linking PD to the risk of colon cancer, six studies linking PD to the risk of rectal cancer, and 12 studies linking PD to the risk of CRC (studies including colon cancer and rectal cancer, but did not analyze it separately). The detailed information of basic characteristics is shown in Table 1.

**TABLE 1:** The basic characteristics of included studies.

Author	Year	Country	Study design	*N* (case)	*M* (%)	Age	*N* (control)	Location	Follow‐up
Powers^13^	2005	USA	Case‐control	217	100.00%	Median: 69	298	Colorectal	NR
Driver^21^	2007	USA	Cohort	487	NR	Median: 72.2	487	Colorectal	5.9‐year
Becker^16^	2010	UK	Cohort	2993	63.30%	NR	3003	Colorectal	NR
Lo^23^	2010	USA	Cohort	692	NR	NR	761	Colorectal	NR
Sun^28^	2011	Taiwan, China	Cohort	4957	51.70%	Mean: 51.7	19,828	Colorectal	NR
Rugbjerg^24^	2012	Denmark	Cohort	20,343	52.70%	Mean: 72.7	32,360	Colorectal	NR
Lin^8^	2015	Taiwan, China	Cohort	62,023	62.60%	Mean: 66	124,046	Colorectal	NR
Boursi^17^	2016	UK	Case‐control	22,093	55.10%	Median: 72	85,833	Colorectal	7‐year
Park^11^	2019	Korea	Cohort	52,009	41.00%	Mean: 71	260,045	Colorectal	NR
Kareus^27^	2012	USA	Cohort	2998	NR	NR	NR	Colorectal	NR
Lu^12^	2021	German	Cohort	328,645	NR	NR	NR	Colorectal	NR
Kim^9^	2023	Korea	Cohort	8381	46.73%	NR	33,524	Colorectal	NR
Olsen^25^	2005	Denmark	Cohort	14,088	NR	Mean: 72.8	NR	Colon +rectum	10‐year
Fois^19^	2009	UK	Cohort	4355	NR	NR	574,860	Colon +rectum	3.4‐year
Ong^5^	2014	UK	Cohort	219,194	57.00%	NR	9,015,614	Colon +rectum	NR
Wirdefeldt^22^	2014	Sweden	Cohort	11,786	60.50%	Mean: 62.5	58,930	Colon +rectum	1‐year
Peretz^26^	2016	Israel	Cohort	7125	54.00%	Mean: 71.2	NR	Colon +rectum	10‐year
Freedman(1)^18^	2016	USA	Case‐control	836,947	54.80%	Mean: 74	142,869	Colon +rectum	5‐year
Guttman^20^	2003	Canada	Cohort	15,304	NR	NR	30,608	Colon	NR
Freedman(2)^29^	2016	Asia	Case‐control	20,267	58.50%	NR	5558	Colon	NR
Agalliu^30^	2019	USA	Cohort	969	61.90%	67.8	218	Colon	NR

Abbreviations: M, male; N, number; NR, not reported.

### 3.2 Quality assessment

Two studies received a score of 5, nine studies received a score of 6, seven studies received a score of 7, and three studies received an 8 when the quality of the included studies was evaluated using the NOS (Table 2).

**TABLE 2:** The quality assessment of included studies.

Selection (0–4) Comparability (0–2) Outcome (0–3)												
Study	REC	SNEC	AE	DO		SC	AF		AO	FU	AFU	Total
Powers^13^	1	1	1			1	1		1			6
Driver^21^	1	1	1			1	1		1			6
Becker^16^	1	1	1			1	1		1			6
Lo^23^	1	1	1			1	1		1	1		7
Sun^28^	1	1	1			1	1		1			6
Rugbjerg^24^	1	1	1			1	1		1	1		7
Lin^8^	1	1	1			1	1		1	1	1	8
Boursi^17^	1	1	1			1	1		1			6
Park^11^	1	1	1			1	1		1	1	1	8
Kareus^27^	1	1	1			1	1		1			6
Lu^12^	1	1	1			1	1		1			6
Kim^9^	1	1	1			1	1		1	1		7
Olsen^25^	1	1	1			1	1		1			6
Fois^19^	1	1	1			1	1		1	1		7
Ong^5^	1	1	1			1	1		1	1	1	8
Wirdefeldt^22^	1	1	1			1	1		1	1		7
Peretz^26^	1	1	1			1	1		1	1		7
Freedman(1)^18^	1	1	1			1	1					5
Guttman^20^	1	1	1			1	1		1			6
Freedman(2)^29^	1	1	1			1	1					5
Agalliu^30^	1	1	1			1	1		1	1		7

Abbreviations: AE, ascertainment of exposure; AF, additional factors; AO, assessment of outcome; AFU, adequacy of follow‐up; DO, outcome not present at the start of the study; FU, length of follow‐up; REC, representativeness of exposed cohort; SNEC, selection of nonexposed cohort; SC, control for important factors.

### 3.3 Relationship between PD and risk of CRC

**FIGURE 2:**
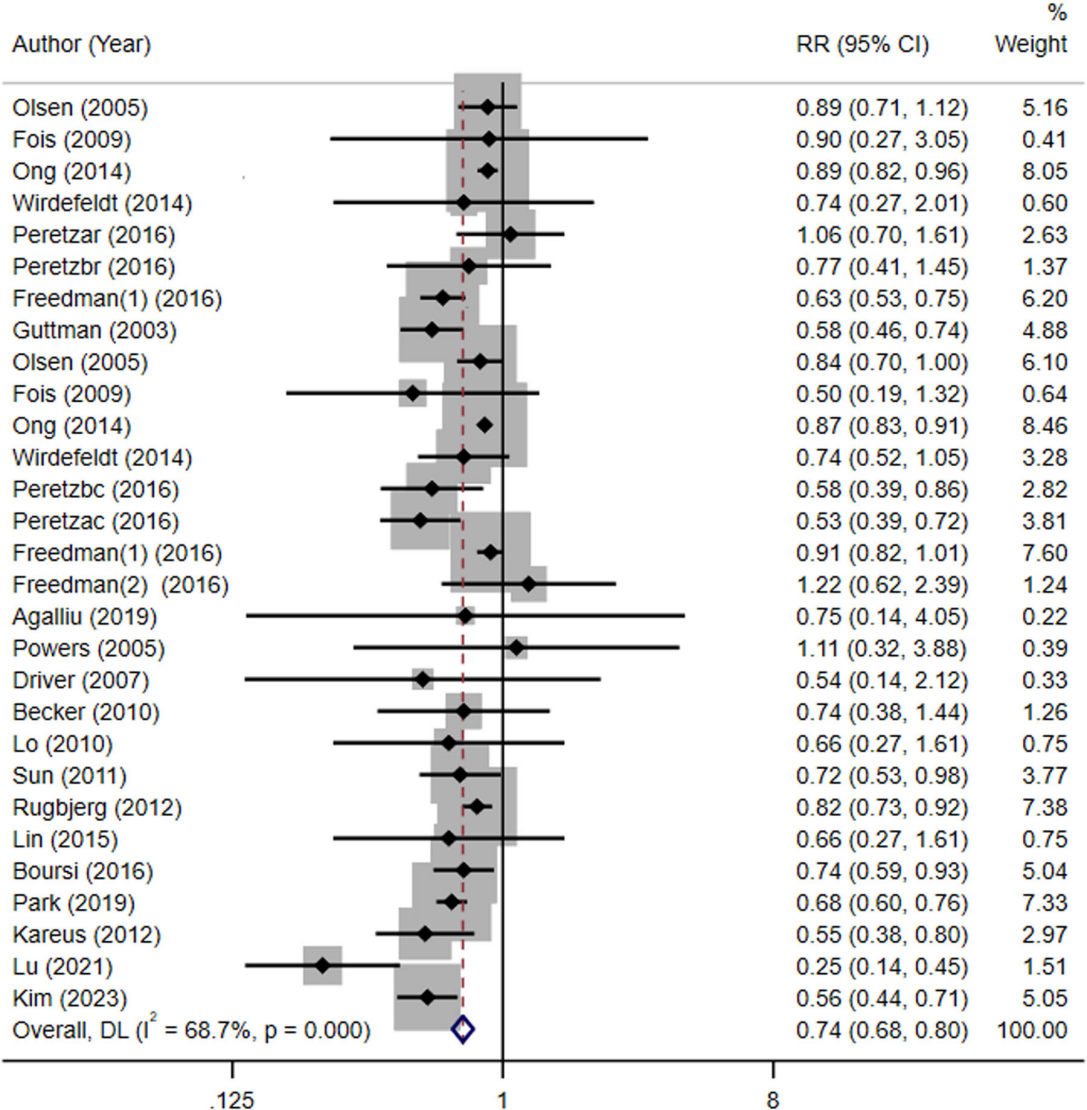
The meta‐analysis forest plot of the association between PD and CRC. Abbreviations: CRC, colorectal cancer; PD, Parkinson's disease; RR, relative risk. [Color figure can be viewed at wileyonlinelibrary.com]

The pooled RRs of CRC in people with and without PD are displayed in Table 3. When all 21 studies were included, we discovered that PD patients had a significantly decreased risk of CRC (RR = 0.74; 95% CI, 0.68–0.80) (Figure 2).

**TABLE 3:** The results of pooled analysis of the outcomes.

	Number of studies	RR	LCI	UCI	*p*	*I* ^2^	*p*
Overall	21	0.74	0.68	0.80	<0.001	68.70%	<0.001
Country							
Asia countries	5	0.67	0.58	0.78	<0.001	28.10%	0.234
Western countries	16	0.76	0.70	0.82	<0.001	64.20%	<0.001
Study design							
Case‐control	4	0.80	0.64	1	0.048	73.40%	0.005
Cohort	17	0.72	0.66	0.79	<0.001	69.10%	<0.001
Location							
Colon	9	0.76	0.67	0.86	<0.001	67.40%	0.001
Rectal	6	0.82	0.69	0.97	0.021	57.70%	0.028
Colorectal	12	0.67	0.57	0.76	<0.001	56.10%	0.009
Year of publication							
Before 2010	5	0.76	0.66	0.88	<0.001	13.90%	0.318
After 2010	16	0.74	0.68	0.80	<0.001	75.90%	<0.001

Abbreviations: LCI, lower confidence interval; RR, relative risk; UCI, upper confidence interval.

### 3.4 Subgroup analysis

Even after the studies were stratified by country, tumor location, and research design, there was still an inverse relationship between CRC risk and PD. The combined risk of PD patients with CRC in Asian nations (RR = 0.67; 95% CI, 0.58–0.78) was lower than that in Western countries (RR = 0.76; 95% CI, 0.70–0.82) (Table 3), according to assessments that were country‐specific. A consistent lower cancer risk was seen in subset analyses of both research types, with a total RR of 0.80 for case‐control studies (RR = 0.80; 95% CI, 0.64–1) and 0.72 for cohort studies (RR = 0.72; 95% CI, 0.66–0.79) (Table 3). As for the tumor location, in comparison to rectal cancer (RR = 0.82; 95% CI, 0.69–0.97), PD patients exhibited a lower combined risk of colon cancer (RR = 0.76; 95% CI, 0.67–0.86) (Table 3). Although the combined risk of PD patients in studies published before 2010 (RR = 0.76; 95% CI, 0.66–0.88) was only slightly higher than that of those in studies published after 2010 (RR = 0.74; 95% CI, 0.68–0.80), they all indicated that PD patients had a significantly decreased risk of CRC. All of these subgroup analyses revealed that the significant inverse association between PD and the risk of CRC was not undermined by many factors, including country, study design, tumor location, and publication time of the study.

### 3.5 Sensitivity analysis and publication bias

**FIGURE 3:**
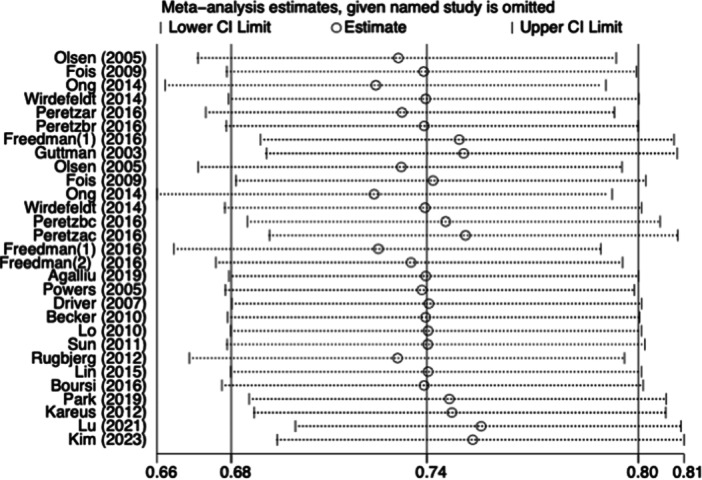
The sensitivity analysis of the association between PD and CRC. Abbreviations: CI, confidence interval; CRC, colorectal cancer; PD, Parkinson's disease.

**FIGURE 4:**
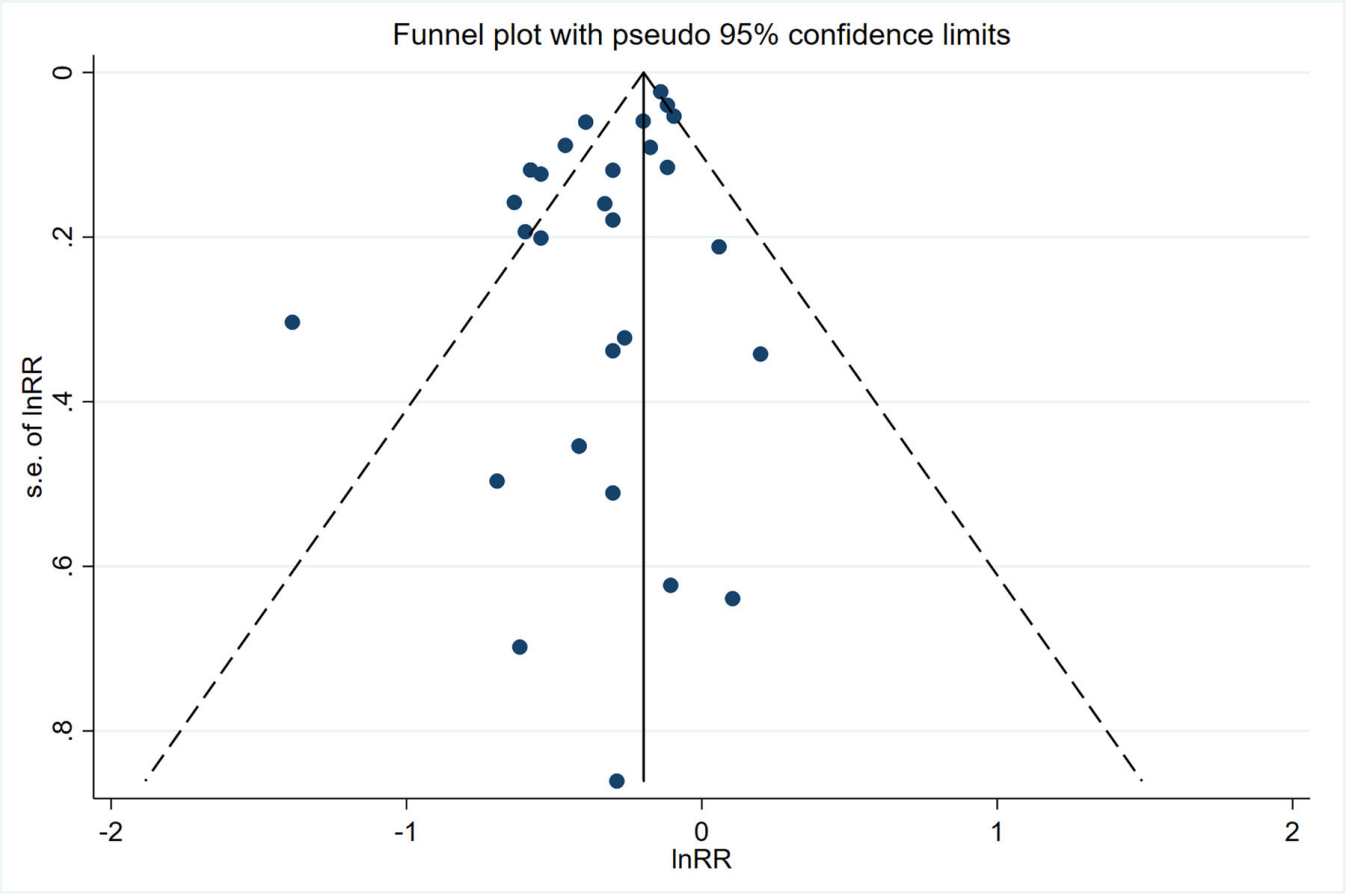
The meta‐analysis funnel plot of the association between PD and CRC. Abbreviations: CRC, colorectal cancer; PD, Parkinson's disease. [Color figure can be viewed at wileyonlinelibrary.com]

Sensitivity analysis revealed no significant variation between studies when the pooled RR and 95% CI were recalculated after omitting each study in turn (Figure 3). Additionally, there was no remarkable publication bias for the risks of CRC based on Begg's test (*p* = 0.91) and inspection of the funnel plot (Figure 4), suggesting that our findings were robust.

## 4 DISCUSSION

PD, a neurodegenerative disorder, and cancer, characterized by uncontrolled cellular proliferation, can be viewed as contrasting biological processes. However, the question arises as to whether there is a close clinical association. Epidemiological evidence demonstrates inconsistent associations between PD and CRC. For instance, one study included 8381 PD patients showed inverse associations between CRC in patients with PD.^9^ Another study, which utilized national cause‐specific death data from 183 countries extracted from the Global Health Observatory database, indicated a negative association between PD and CRC.^12^ Furthermore, a meta‐analysis investigating the association between PD and cancer revealed that individuals with PD exhibited decreased susceptibility to colon, rectal, colorectal, and lung cancers while experiencing heightened risks of brain cancer and melanoma.^31^ However, a third study utilizing Epidemiology, Surveillance, and End Results‐Medicare linked data (1992–2005) of persons over 65 years, which included 743,779 cancer patients with PD as well as a non‐cancer group (*n* = 419,432) in prospective cohort analyses, showed no correlation between CRC risk and PD.^18^ Thus, it was necessary to comprehensively analyze the complex associations between the two conditions in a large population. So far, our study was the largest meta‐analysis to assess the relationship between CRC and PD. Although inconsistent results existed in some research studies, the investigation including 21 studies involving 1,635,873 PD patients and 10,388,842 healthy individuals indicated that PD is a protective factor against CRC. Besides, we carried out subgroup analysis using stratified variables such as tumor location, research design, research region, and publication time of the study, which all support a negative association between PD and the risk of CRC. Based on the above results, we can conclude that PD patients might have a lower risk of CRC.

Several mechanisms contribute to the pathogenesis of PD and some kinds of neoplasms, including mitochondrial dysfunction, oxidative stress, DNA damage, abnormalities in mitosis‐stimulating signals, inflammatory factors, and anomalies in cell cycle activation. Additionally, certain biochemical substances, such as α‐synuclein, a marker for PD, may potentially stimulate malignant cells.^32^ Several studies suggest a potential association between dopamine receptors (DR) and susceptibility to malignancies. DR polymorphisms have been linked to an increased risk of non‐small‐cell lung cancer and gastric cancer, while heightened expression of dopamine D2 receptors (DR2) has been observed in various cancer types such as gastric cancer, neuroendocrine tumors, glioma, and breast cancer.^33^ One study reported several genes that link the association between cancer and PD. Approximately half of the cases of PD with early onset are caused by mutations in the Parkin gene, which was already thought to be a tumor suppressor gene due to its location on the long arm of chromosome 6, a section of which has long been recognized to be changed or deleted in a variety of human malignancies. Furthermore, parkinsonism‐associated deglycase (PARK7), and leucine‐rich repeat kinase 2 (LRRK2) play a vital role in the link between the two diseases.^34^ Additionally, Chen and his colleagues reported that PD patients had decreased probabilities of developing colon cancer, owing to the regulation of autophagy flux by the PD‐associated gene ATPase cation transporting 13A2 (ATP13A2).^35^ Additionally, one research found that dysbiosis and changed colonic microbiota in PD patients may be the mechanism behind the association between CRC and PD.^36^ Previous research has presented that the fecal microbiota and colonic mucosa from patients with PD differ from those of healthy people.^37^ Other mechanisms of the pathogenesis of PD and neoplasms, such as oxidative stress, mitochondrial dysfunction, inflammatory factors, DNA damage, and abnormal mitosis‐stimulating signals also play critical roles, which still need further investigation.

The strengths of this meta‐analysis and systematic review include that it utilizes the largest clinical data to assess the correlation between CRC risk and PD. In recent years, an increasing number of studies have explored this correlation; this work provides a comprehensive summary, stratified by year of publication. Although previous research presented conflicting views on the connection between CRC risk and PD, this review, with the largest sample size available, indicated that PD is associated with a lower risk of CRC.

However, there are limitations to this study. The included investigations were predominantly retrospective, introducing potential bias. While efforts were made to match the most significant confounding variables, and multivariate analyses were conducted to obtain reliable estimates of HR, OR, and RR, some factors may still not have been accounted for. Additionally, the funnel plot analysis revealed that a substantial number of studies fell outside the CIs, particularly at the top of the funnel. This suggests that the inclusion of a larger number of studies may compromise the unbiased conclusion due to increased variability in estimated effect sizes. This variability could be attributed to differences in patient enrollment criteria, such as age, race, sex, and individual patient variation. What is more, the etiology of CRC and PD, as well as the underlying mechanisms behind the inverse association between these two diseases, are complex and warrant further investigation.

Building upon the current study, future research could focus on the underlying mechanism of decreased risk of CRC in PD patients. Emerging research supports the existence of shared genetic pathways between PD and cancer, offering potential insights for novel approaches to tumor management. Furthermore, advancing understanding of the etiopathogenesis of PD and cancer has the potential to facilitate the creation of innovative diagnostic techniques with practical implications. Elucidating the potential mechanisms underlying these pathologies and their interrelated dependencies may serve as a foundation for the development of precise therapeutic interventions for both disease conditions.

## 5 CONCLUSION

According to our results, patients with PD had a lower risk of CRC; however, the underlying mechanisms are still unclear and require further research to be fully understood.

## AUTHOR CONTRIBUTION

Meng‐Dan Su and Fei Liu conceptualized the study; Meng‐Dan Su, Hao‐Wen Zhang, Ke‐Yu Cao, Tian‐Hong Wang, and Fei Liu developed the methodology; Meng‐Dan Su and Hao‐Wen Zhang managed the software; Meng‐Dan Su, Hao‐Wen Zhang, and Tian‐Hong Wang conducted the statistical analysis and revised the manuscript; Meng‐Dan Su, Hao‐Wen Zhang, and Fei Liu reviewed the manuscript. All the authors have read and approved the final version of the manuscript.

## CONFLICT OF INTEREST STATEMENT

Fei Liu, who is also an editorial member of Ibrain, is excluded from editorial decision‐making related to the acceptance and publication of this article. Editorial decision‐making was handled independently by the editors‐in‐chief to minimize bias. Other authors declare no conflicts of interest.

## ETHICS STATEMENT

Not applicable since all analyses were based on previously published research.

## Data Availability

The data supporting this study are available on request from the corresponding author.

## References

[1] Bloem BR , Okun MS , Klein C . Parkinson's disease. Lancet. 2021;397(10291):2284‐2303. 10.1016/s0140-6736(21)00218-x 33848468

[2] Dorsey ER , Elbaz A , Nichols E , et al. Global, regional, and national burden of Parkinson's disease, 1990–2016: a systematic analysis for the Global Burden of Disease Study 2016. Lancet Neurol. 2018;17(11):939‐953. 10.1016/s1474-4422(18)30295-3 30287051 PMC6191528

[3] Qi S , Yin P , Wang L , et al. Prevalence of Parkinson's disease: a community‐based study in China. Mov Disorders. 2021;36(12):2940‐2944. 10.1002/mds.28762 34390510

[4] Jankovic J . Parkinson's disease: clinical features and diagnosis. J Neurol Neurosurg Psychiatry. 2008;79(4):368‐376. 10.1136/jnnp.2007.131045 18344392

[5] Ong EL , Goldacre R , Goldacre M . Differential risks of cancer types in people with Parkinson's disease: a national record‐linkage study. Eur J Cancer. 2014;50(14):2456‐2462. 10.1016/j.ejca.2014.06.018 25065294

[6] Hang Z , Lei T , Zeng Z , Cai S , Bi W , Du H . Composition of intestinal flora affects the risk relationship between Alzheimer's disease/Parkinson's disease and cancer. Biomed Pharmacother = Biomed Pharmacother. 2022;145:112343. 10.1016/j.biopha.2021.112343 34864312

[7] Leong YQ , Lee SWH , Ng KY . Cancer risk in Parkinson disease: an updated systematic review and meta‐analysis. Eur J Neurol. 2021;28(12):4219‐4237. 10.1111/ene.15069 34403556

[8] Lin PY , Chang SN , Hsiao TH , Huang BT , Lin CH , Yang PC . Association between Parkinson disease and risk of cancer in Taiwan. JAMA Oncol. 2015;1(5):633‐640. 10.1001/jamaoncol.2015.1752 26181771

[9] Kim SY , Choi HG , Kim YH , et al. Longitudinal study of the inverse relationship between Parkinson's disease and cancer in Korea. npj Parkinson's Dis. 2023;9(1):116. 10.1038/s41531-023-00562-5 37481603 PMC10363116

[10] Morgan E , Arnold M , Gini A , et al. Global burden of colorectal cancer in 2020 and 2040: incidence and mortality estimates from GLOBOCAN. Gut. 2023;72(2):338‐344. 10.1136/gutjnl-2022-327736 36604116

[11] Park JH , Kim DH , Park YG , et al. Cancer risk in patients with Parkinson's disease in South Korea: a nationwide, population‐based cohort study. Eur J Cancer. 2019;117:5‐13. 10.1016/j.ejca.2019.04.033 31229950

[12] Lu L , Dai M , Mullins CS , Schafmayer C , Linnebacher M . Global association of cause‐specific mortality between the major gastrointestinal cancers and Parkinson's disease for the first two decades of the new millennium. Aging Dis. 2022;13(2):534‐539. 10.14336/ad.2021.1016 35371614 PMC8947825

[13] Powers KM , Smith‐Weller T , Franklin GM , Longstreth Jr. WT , Swanson PD , Checkoway H . Diabetes, smoking, and other medical conditions in relation to Parkinson's disease risk. Parkinsonism Rel Disord. 2006;12(3):185‐189. 10.1016/j.parkreldis.2005.09.004 16364673

[14] Page MJ , McKenzie JE , Bossuyt PM , et al. The PRISMA 2020 statement: an updated guideline for reporting systematic reviews. BMJ. 2021;372:n71. 10.1136/bmj.n71 33782057 PMC8005924

[15] Stang A . Critical evaluation of the Newcastle‐Ottawa scale for the assessment of the quality of nonrandomized studies in meta‐analyses. Eur J Epidemiol. 2010;25(9):603‐605. 10.1007/s10654-010-9491-z 20652370

[16] Becker C , Brobert GP , Johansson S , Jick SS , Meier CR . Cancer risk in association with Parkinson disease: a population‐based study. Parkinsonism Rel Disord. 2010;16(3):186‐190. 10.1016/j.parkreldis.2009.11.005 19945903

[17] Boursi B , Mamtani R , Haynes K , Yang YX . Parkinson's disease and colorectal cancer risk‐a nested case control study. Cancer Epidemiol. 2016;43:9‐14. 10.1016/j.canep.2016.05.007 27232063 PMC4963291

[18] Freedman DM , Wu J , Chen H , et al. Associations between cancer and Parkinson's disease in U.S. elderly adults. Int J Epidemiol. 2016;45(3):741‐751. 10.1093/ije/dyw016 26989123 PMC5841885

[19] Fois AF , Wotton CJ , Yeates D , Turner MR , Goldacre MJ . Cancer in patients with motor neuron disease, multiple sclerosis and Parkinson's disease: record linkage studies. J Neurol Neurosurg Psychiatry. 2010;81(2):215‐221. 10.1136/jnnp.2009.175463 19726405

[20] Guttman M , Slaughter PM , Theriault ME , DeBoer DP , Naylor CD . Parkinsonism in Ontario: comorbidity associated with hospitalization in a large cohort. Mov Disorders. 2004;19(1):49‐53. 10.1002/mds.10648 14743360

[21] Driver JA , Logroscino G , Buring JE , Gaziano JM , Kurth T . A prospective cohort study of cancer incidence following the diagnosis of Parkinson's disease. Cancer Epidemiol Biomarkers Prevent: Publ Am Assoc Cancer Res Cosponsored Am Soc Prevent Oncol. 2007;16(6):1260‐1265. 10.1158/1055-9965.Epi-07-0038 17548694

[22] Wirdefeldt K , Weibull CE , Chen H , et al. Parkinson's disease and cancer: a register‐based family study. Am J Epidemiol. 2014;179(1):85‐94. 10.1093/aje/kwt232 24142916 PMC3864714

[23] Lo RY , Tanner CM , Van Den Eeden SK , Albers KB , Leimpeter AD , Nelson LM . Comorbid cancer in Parkinson's disease. Mov Disorders. 2010;25(12):1809‐1817. 10.1002/mds.23246 20669266

[24] Rugbjerg K , Friis S , Lassen CF , Ritz B , Olsen JH . Malignant melanoma, breast cancer and other cancers in patients with Parkinson's disease. Int J Cancer. 2012;131(8):1904‐1911. 10.1002/ijc.27443 22278152 PMC3636769

[25] Olsen JH , Friis S , Frederiksen K , McLaughlin JK , Mellemkjaer L , Møller H . Atypical cancer pattern in patients with Parkinson's disease. Br J Cancer. 2005;92(1):201‐205. 10.1038/sj.bjc.6602279 15583688 PMC2361753

[26] Peretz C , Gurel R , Rozani V , et al. Cancer incidence among Parkinson's disease patients in a 10‐yrs time‐window around disease onset: a large‐scale cohort study. Parkinsonism Rel Disord. 2016;28:68‐72. 10.1016/j.parkreldis.2016.04.028 27161827

[27] Kareus SA , Figueroa KP , Cannon‐Albright LA , Pulst SM . Shared predispositions of parkinsonism and cancer: a population‐based pedigree‐linked study. Arch Neurol. 2012;69(12):1572‐1577. 10.1001/archneurol.2012.2261 22945795

[28] Sun LM , Liang JA , Chang SN , Sung FC , Muo CH , Kao CH . Analysis of Parkinson's disease and subsequent cancer risk in Taiwan: a nationwide population‐based cohort study. Neuroepidemiology. 2011;37(2):114‐119. 10.1159/000331489 21986194

[29] Freedman DM , Pfeiffer RM . Associations between Parkinson disease and cancer in US Asian Americans. JAMA oncol. 2016;2(8):1093‐1094. 10.1001/jamaoncol.2016.0729 27196082

[30] Agalliu I , Ortega RA , Luciano MS , et al. Cancer outcomes among Parkinson's disease patients with leucine rich repeat kinase 2 mutations, idiopathic Parkinson's disease patients, and nonaffected controls. Mov Disorders. 2019;34(9):1392‐1398. 10.1002/mds.27807 PMC6754269 31348549

[31] Lee JYS , Ng JH , Saffari SE , Tan EK . Parkinson's disease and cancer: a systematic review and meta‐analysis on the influence of lifestyle habits, genetic variants, and gender. Aging. 2022;14(5):2148‐2173. 10.18632/aging.203932 35247252 PMC8954974

[32] Ejma M , Madetko N , Brzecka A , et al. The links between Parkinson's disease and cancer. Biomedicines. 2020;8(10):416. 10.3390/biomedicines8100416 33066407 PMC7602272

[33] Wang X , Wang ZB , Luo C , et al. The prospective value of dopamine receptors on bio‐behavior of tumor. J Cancer. 2019;10(7):1622‐1632. 10.7150/jca.27780 31205518 PMC6548012

[34] Garber K . Parkinson's disease and cancer: the unexplored connection. J Natl Cancer Inst. 2010;102(6):371‐374. 10.1093/jnci/djq081 20215596

[35] Chen Q , Zhong L , Zhou C , et al. Knockdown of Parkinson's disease‐related gene ATP13A2 reduces tumorigenesis via blocking autophagic flux in colon cancer. Cell Biosci. 2020;10(1):144. 10.1186/s13578-020-00506-z 33308286 PMC7731751

[36] Keshavarzian A , Green SJ , Engen PA , et al. Colonic bacterial composition in Parkinson's disease. Mov Disorders. 2015;30(10):1351‐1360. 10.1002/mds.26307 26179554

[37] Tsao SP , Nurrahma BA , Kumar R , et al. Probiotic enhancement of antioxidant capacity and alterations of gut microbiota composition in 6‐hydroxydopamin‐induced Parkinson's disease rats. Antioxidants. 2021;10(11):1823. 10.3390/antiox10111823 34829694 PMC8615185

